# Silicone Facial Implants, to Fixate or Not to Fixate: A Narrative Review

**DOI:** 10.7759/cureus.34524

**Published:** 2023-02-01

**Authors:** Mohamed Gafar Ahmed, Ziyad A AlHammad, Badr Al-Jandan, Turki Almohammadi, Mohammad Khursheed Alam, Hiroj Bagde

**Affiliations:** 1 Department of Oral and Maxillofacial Surgery, King Fahd Hospital of the University, Dammam, SAU; 2 Saudi Board of Oral & Maxillofacial Surgery, Ministry of Health, Riyadh, SAU; 3 Department of Biomedical Dental Sciences, College of Dentistry, Imam Abdulrahman Bin Faisal University, Dammam, SAU; 4 Department of Oral and Maxillofacial Surgery, College of Dentistry, Jouf University, Sakaka, SAU; 5 Department of Preventive Dentistry, College of Dentistry, Jouf University, Sakaka, SAU; 6 Department of Periodontology, Rama Dental College Hospital and Research Centre, Kanpur, IND

**Keywords:** augmentation., complications, stabilization, chin, angle, malar, implant fixation, facial implants, silicone

## Abstract

Silicone implants are one of the most widely used implants for facial augmentation, especially in the chin, mandibular angle, and malar area, utilizing different surgical approaches. Despite their various advantages, many complications have also been reported, including hematoma, infection, bone resorption, numbness, displacement, and asymmetry. This study aims to evaluate the need for facial-implant fixation and compare and contrast fixated and nonfixated facial silicone implants in different facial sites. A narrative review of the topic of facial-implant stabilization using the PubMed database inclusion criteria included articles that discussed the topic of facial implants, were published in English, and included critical information such as the location of the implant, type of stabilization, follow-up periods, and complications. A total of 11 studies were included. Of these, two were prospective clinical studies, three were case series, and the remaining six were retrospective clinical studies. The studies were published between 1995 and 2018. The sample size varied from 2 to 601 cases. Stabilization includes suturing, monocortical screws, or no stabilization. Complications were reported in most of these studies, including asymmetry, bone resorption or erosion, displacement, dissatisfaction, edema, hematoma, infection, mucosal irritation, pain, and paresthesia. The follow-up period ranged from one month to 17 years. Despite the varied settings of these studies, silicone facial implant complications were reported in both fixated and nonfixated implants, with a lack of significant differences between fixated and nonfixated facial silicone implants regarding the method of fixation.

## Introduction and background

Facial implants are used for various purposes, such as the reconstruction of post-traumatic defects, addressing developmental and congenital abnormalities, and, most commonly, improving aesthetics [1]. Common locations of aesthetic concern include the malar region, chin, and mandibular angle [2]. Advanced facial-implant materials have been the trend recently over traditional autogenous bone grafts as they offer numerous advantages, such as decreased donor site morbidity, elimination of graft resorption, and increased implant availability [3].

Silicone elastomer is a solid, rubber-consistency polymer made of polydimethylsiloxane. Due to its relative inertness as an implant, it has been developed for many medical applications, particularly facial-skeletal augmentation [4]. The use of dimethylsiloxane (silicone) is widespread throughout many areas of medicine and surgery and is associated with a remarkable paucity of significant adverse tissue reactivity. It is used in the face primarily as an onlay implant for the reconstruction of zygomatic, maxillary, nasal, and mandibular contours [5]. Although alloplastic silicone is becoming one of the most commonly used materials for facial implants, it still carries its own set of complications. Alloplastic facial-implant complications include bone resorption, erosion, infection, mucosal irritation, hematoma, edema, pain, numbness, displacement, asymmetry, and dissatisfaction [6]. Different complications were reported in the literature at various implant sites and utilizing various stabilization techniques [7]. Malar augmentation with the Silastic midfacial malar implant is a reliable and effective means of correcting many types of malar defects. In a retrospective study by Metzinger et al., 60 patients with complaints of malar hypoplasia or facial asymmetry underwent Silastic midfacial malar implants with no means of fixation. After a two-year follow-up, they reported four cases of superior displacement and one of initial misalignment requiring revision [8].

Using lateral chin radiographs, Sciaraffia et al. evaluated the presence of bone resorption after the insertion of silicone chin implants. Fourteen patients presented with bone erosion, with a maximum of 2.0 mm of erosion, after a one-year follow-up [9]. The mandibular angle is an essential part of the skeletal framework; therefore, its augmentation plays an important role in maintaining and improving facial aesthetics [10]. In a previous study involving a prospective analysis of the outcomes of mandibular angle region augmentation with bilateral silicone implants, the overall incidence of implant displacement was 13.8% [11]. Facial implants should be immobilized. While it is not a common practice to stabilize the implant by suturing it to surrounding soft tissues or by using temporary transcutaneous pull-out sutures [12], other authors suggest screw fixation of the implant to the underlying bone, in addition to implant immobilization, which creates holes between the implant and the underlying bone and possibly improves augmentation [13]. On the other hand, facial-implant placement without stabilization by either sutures or screws was also reported in some literature [14].

The current literature shows a lack of evidence with respect to alloplastic facial implant stabilization. So, the goal of this study is to figure out if stabilization of facial implants is needed and how it affects complications with facial implants.

## Review

A narrative review of articles addressing the topic of facial implant stabilization was conducted. The review was carried out in March 2020 via searches of the PubMed database for specific keywords: silicone, facial implants, malar, angle, chin, stabilization, and complications. Inclusion criteria comprised articles that discussed the topic of facial implants, with an emphasis on the placement of silicone implants in the chin area, malar areas, and mandibular angles, and studies reporting rates of complications involving fixated and nonfixated silicone implants. All included articles were PubMed indexed, published in English, and reported on critical information, such as the location of the implant, type of stabilization, and complications. Exclusion criteria were applied to exclude the following: new operative techniques, animal studies, studies of silicone-implant stabilization with no follow-up details, and nonimplant or surgical complications. A total of 153 articles were reviewed. Of the studies analyzed, only six retrospective clinical studies, two prospective clinical studies, and three case series were included. Case series were included due to the limited amount of literature emphasizing the subject of silicone-implant fixation and because the case series addressed critical complications.

The years of publication ranged from 1995 to 2018. The number of patients included ranged in each study from 2 to 601 patients, for a total of 1,051 patients. The total number of patients who had fixated implants was 799 (76.02%) [11,12,15,16], and 252 patients (23.97%) had nonfixated implants [8,9,14,17-20]. The number of implants based on implant site was 933 in the chin (81.05%) [9,12,14-20], 160 in the malar region (13.90%) [8,16], and 58 (5.03%) as angle implants [11]. Follow-up periods ranged from one month to 17 years, except for two case series studies where follow-up periods were not reported [18,19]. The reported complications include asymmetry, bone resorption or erosion, displacement, dissatisfaction, edema, hematoma, infection, mucosal irritation, pain, and paresthesia. The publication author, year of publication, type of study, number of cases, location of the implant, type of fixation, follow-up period, and reported complications are given in Table 1.

**Table 1:** Summary of 11 included articles. *N*, number

Author	Year	Study Design	Patients no.	Location	Stabilization	Follow-up	Complications (N) %
Fixated (Stabilized)							
Al-Jandan and Marei [11]	2018	Prospective	58	Angle	Two monocortical screws of 2 mm × 7 mm	1 year	Displacement, (8) 13.8%; dissatisfaction: (3) 5.1%; infection: (3) 5.2%
Pitanguy et al. [15]	1986	Prospective	601	Chin	Sutured to orbicularis oris muscle	16 years	Displacement, (4) 0.66%; dissatisfaction, (1) 0.16%
Vuyk [12]	1996	Retrospective	40	Chin	Suture: 4-0 Proline	1-45 months	Asymmetry, (3) 7.5%; bone resorption, (8) 20%; and hematoma, (2) 5%
Hopping et al. [16]	2010	Retrospective	100	Chin Malar	Suture: Chin: 4-0 Vicryl Malar: 4-0 Nylon	4 years	Chin: dissatisfaction (1) 1% and pain (1) 1% Malar: asymmetry (2) 2%; infection (8) 8%
Nonfixated (Nonstabilized)							
Aynehchi et al. [14]	2011	Retrospective	125	Chin	None	1 year	Mucosal irritation (2) 1.6%
Sciaraffia et al. [9]	2018	Retrospective	15	Chin	None	1 year	Bone erosion: 0 mm erosion: (1) 6.7% < 1.5 mm erosion: (11) 73.3% > 1.5 mm erosion: (3) 20%
Metzinger et al. [8]	1999	Retrospective	60	Malar	None	1 year	Displacement (5) 8.3%; dissatisfaction (1) 1.7%; edema (1) 1.7%; paresthesia (3) 5%
Saleh et al. [17]	2002	Retrospective	40	Chin	None	9-60 months	Bone resorption: 0.5 mm, (14) 35%; 1 mm, (3) 7.5%; and 2 mm (4) 10%
Abrahams and Caceres [18]	1997	Case series	4	Chin	None	Not reported	Bone erosion: < 3 mm, (1) 25%; 3 mm in (2) 50% > 3 mm in (4) 25%
Matarasso et al. [19]	1995	Case series	6	Chin	None	Not reported	Bone erosion: < 5 mm, (1) 17%; 5-10 mm, (3) 50% > 10 mm, (2) 33%
Polo [20]	2016	Case series	2	Chin	None	10-17 years	Bone erosion: first patient, none; second patient, 0.5 mm

Fixated facial implants and complications

The total number of patients with fixated (stabilized) implants was 799 (76.02%) [11,12,15,16]. The average complication rate with fixated facial implants was 5.5%. The most common complications associated with fixated-facial implants were displacement (2.5%), followed by infection (1.37%), bone resorption or erosion (1%), asymmetry (0.62%), dissatisfaction (0.62%), hematoma (0.25%), and pain (0.12%).

Nonfixated facial implants and complications

The total number of patients with nonfixated (nonstabilized) implants was 252 (23.97%) [8,9,14,17-20]. The average complication rate with nonfixated facial implants was 23.41%. The most common complication associated with nonfixated facial implants was bone resorption (18.25%), followed by displacement (1.98%), paresthesia (1.19%), mucosal irritation (0.79%), dissatisfaction (0.39%), and edema 0.39% (Table 2).

**Table 2:** Complications in relation to implant stabilization.

Fixation	Fixated implants	Nonfixated implants	Total
Total number of patients	799	252	1,051
Average complication rates (%)			
Asymmetry	(5) 0.62%	(0) 0.00%	(5) 0.47%
Bone resorption/erosion	(8) 1%	(46) 18.25%	(54) 5.13%
Displacement	(12) 2.5%	(5) 1.98%	(17) 1.61
Dissatisfaction	(5) 0.62%	(1) 0.39%	(6) 0.57%
Edema	(0) 0.00%	(1) 0.39%	(1) 0.09%
Hematoma	(2) 0.25%	(0) 0.00%	(2) 0.19%
Infection	(11) 1.37%	(0) 0.00%	(11) 1.04%
Mucosal irritation	(0) 0.00%	(2) 0.79%	(2) 0.19%
Pain	(1) 0.12%	(0) 0.00%	(1) 0.09%
Paresthesia	(0) 0.00%	(3) 1.19%	(3) 0.28%
Total average complication rate	(44) 5.5%	(58) 23.41%	(102) 9.69%

Chin implant and complications

The total number of chin implants was 933 (81.05%) [9,12,14-20]. The average complication rate with chin implants was 7.2%. The most common complication associated with chin implants was bone resorption or erosion (5.78%), followed by displacement (0.42%), asymmetry (0.32%), dissatisfaction (0.21%), hematoma (0.21%), mucosal irritation (0.21%), and pain (0.1%). 

Malar implant and complications

The total number of malar implants was 160 (13.90%) [8,16]. The average complication rate with chin implants was 7.2%. The most common complication associated with chin implants was bone resorption or erosion (5.78%), followed by displacement (0.42%), asymmetry (0.32%), dissatisfaction (0.21%), hematoma (0.21%), mucosal irritation (0.21%), and pain (0.1%).

Angle implant and complications

The total number of angle implants was 58 (5.03%) [11]. The average complication rate with chin implants was 24.13%. The most common complication associated with chin implants was displacement (13.79%), followed by dissatisfaction (5.17%), and infection (5.17%) (Table 3).

**Table 3:** Complications with respect to the implant location.

Location	Chin	Malar	Angle
Total number of implants	933	160	58
Average complication rates (%)			
Asymmetry	(3) 0.32%	(2) 1.25%	(0) 0.00%
Bone resorption/erosion	(54) 5.78%	(0) 0%	(0) 0.00%
Displacement	(4) 0.42%	(5) 3.12%	(8) 13.79%
Dissatisfaction	(2) 0.21%	(1) 0.62%	(3) 5.17%
Edema	(0) 0%	(1) 0.62%	(0) 0.00%
Hematoma	(2) 0.21%	(0) 0%	(0) 0.00%
Infection	(0) 0%	(8) 5%	(3) 5.17%
Mucosal irritation	(2) 0.21%	(0) 0%	(0) 0.00%
Pain	(1) 0.10%	(0) 0%	(0) 0.00%
Paresthesia	(0) 0%	(3) 1.87%	(0) 0.00%
Total average complication rate	(68) 7.2%	(20) 12.5%	(14) 24.13%

Prevalence of fixated versus nonfixated facial implants

The amount of published literature addressing nonfixated facial implants is higher than that of fixated implants; this could be attributed to multiple factors, such as the ease of use of nonfixated implants, their cost, and the surgeon’s preference. It is evident that there are more studies on fixated silicone implants utilizing suturing techniques than utilizing hardware (3:1) [11,12,15,16]; this could be influenced by surgical skills, knowledge, and comfort with using hardware in various transoral approaches compared to using suturing techniques. Moreover, fixing a facial implant using hardware requires more surgical preparation, setup, and modifications, including the type of anesthesia, operative armamentarium, and duration of the procedure. This has a significant financial impact on the overall procedure [10,11,13]. All of these factors could lead to the decision not to use hardware to fixate facial implants, which is reflected in the relatively low number of studies involving hardware.

Overall complications of fixated versus nonfixated facial implants

When comparing overall complications in fixated and nonfixated implants, it was found that there was an overall complication rate of 5.5% with fixated implants in 799 patients and a complication rate of 23.41% in 252 patients with nonfixated implants, for a total of 9.69% as the overall complication rate of the 1,051 patients. This difference in overall complication rates is in favor of implant stabilization. Fixated cases outnumbered the nonfixated cases because one study by Pitanguy et al. had a disproportionately high number of cases: 601, in which suturing techniques were applied were studied over 16 years of follow-up [15].

Displacement, asymmetry, and dissatisfaction

Comparing asymmetry and dissatisfaction between studies, stabilized implants showed relatively similar incidences of asymmetry (0.62%) and dissatisfaction (0.39%) compared to nonstabilized implants. This could be attributed to a lack of data, as only one included the study of nonstabilized facial implants, which reported the incidence of asymmetry and dissatisfaction [8] compared to three studies of stabilized facial implants [11,12,16]. Nevertheless, asymmetry was mainly reported in the chin (0.32%) [12] and malar [16] (1.25%), and dissatisfaction was more significant in the angle (5.17%) [11]. It is important to differentiate between displacement and asymmetry. Displacement indicates a postoperative complication of implant migration from its original site, whereas asymmetry reflects the improper placement of the implant about the contralateral side or facial profile. Both can occur due to improper implant size selection, wide surgical access, over-dissection, or under-dissection. Displacement was reported in three studies. Al-Jandan and Marei reported the incidence of screw-stabilized implant displacement in the angle area to be 13.8% [11]. Pitanguy et al. reported the incidence of suture-stabilized implant displacement in the chin area to be 0.66% [15]. Metzinger et al. reported the incidence of nonstabilized implant displacement in the malar area to be 8.3% [8]. Such findings indicate that the location of the implant might play an important role. Whether hardware or sutures were used to fixate implants, there is a considerable rate of displacement: 13.8% displacement in screw-fixed implants [11] and 0.66% displacement in sutured implants [15]. Unfortunately, these statistics do not portray the effect of using hardware on the stability of implants compared to suture stabilization as more studies in hardware fixation are required to demonstrate significant relationships between hardware fixation and displacement in comparison with suturing techniques.

Hematoma, infection, and edema

Abnormal fluid collections such as hematoma and seroma can result from inadequate hemostasis, over-dissection, traumatic handling of the tissues, dead space around or underneath the implant, or elevated blood pressure [12]. Hematomas and seromas encourage the growth of bacterial contamination, potentiating cellulitis and infection. They can result in excessive fibrosis-producing soft tissue defects [11,13]. Theoretically, fixing implants to the underlying bone would result in increasing the contact surface between underlying hard tissues and the implant, resulting in fewer voids and dead spaces and subsequently reducing the chances of fluid collection underneath implants and infection. However, this is not reflected in the results of our study, where fixated-implant cases had hematomas 0.25% of the time [12], compared to 0% in nonfixated implants. This could reflect the extent of tissue manipulation and handling during the fixation of implants, in contrast to the minimal tissue handling in the nonfixated cases. Infection was reported in only 11 cases of fixated implants, with a prevalence of 1.37% [11,16]. Regardless of the use or nonuse of fixation, infection was mainly reported in the mandibular angles at 5.17% [11] and malar areas at 5% [16]. On the other hand, postoperative edema was recorded in one case with nonstabilized implants at 0.39% [8]. In general, there is not sufficient data to list the causes of infection per case or explain the low percentage of edema.

Bone resorption or erosion

Theoretically, bone resorption is minimized when implants are immobilized and positioned in a supraperiosteal or subperiosteal plane. Wellisz et al. compared resorptive changes with different implant materials and demonstrated that all nonfixated implants have the potential to result in moderate-to-severe resorption [21]. Pearson and Sherris concluded that implant position in relation to the periosteum did not appear to correlate with bone resorption. Furthermore, they discovered that increasing the implant's pressure against the underlying bone may reduce resorption, which is supported by evidence that bone responds to mechanical stress by deposition of mineralized bone at compression sites and resorption at stress-free sites [22]. Some authors suggest that the probable reason for bone resorption might be the fact that implants are not stabilized with screws, which can lead to continuous micromovement and consequently to bone resorption [23-27]. Mobility of the implant and the resulting pressure on the underlying bone increase bone resorption; thus, screw fixation limits implant mobility and its consequences [28-31]. This supports the theory that implant fixation could decrease the degree of bone resorption compared to nonfixated implants.

Pain and paresthesia

Regarding pain and paresthesia, they were considered in only two studies. One study by Hopping et al. reported persistent pain in suture-stabilized chin implants (1%) [16]. One study by Metzinger et al. reported paresthesia of nonstabilized malar implants (5%) [8]. A reasonable explanation for these complications could be the closeness of the mental nerve to the chin area and the infraorbital nerve to the malar area.

## Conclusions

Silicone is one of the most frequently used materials for facial implants, given its many advantages. However, infection, edema, hematoma, pain, paresthesia, displacement, and bone resorption have all been reported in both stabilized and nonstabilized implants. While one technique has not favored over the other, surgical skills and experience play the most significant role in a successfully placed facial implant. As the current literature lacks significant evidence regarding silicone-implant stabilization, a direct correlation between implant fixation and its complications cannot be established. However, the authors of this study prefer to stabilize silicone implants with titanium monocortical screws to achieve better results and hypothetically reduce postoperative complications. For stronger proof, we need more controlled clinical trials, investigations, and studies with long-term follow-ups. 

## References

[1] Frodel JL, Lee S (1998). The use of high-density polyethylene implants in facial deformities. Arch Otolaryngol Head Neck Surg.

[2] Cuzalina LA, Hlavacek MR (2009). Complications of facial implants. Oral Maxillofac Surg Clin North Am.

[3] Al-Khdhairi OB, Abdulrazaq SS (2017). Is orbital floor reconstruction with titanium mesh safe?. J Craniofac Surg.

[4] Mojsiewicz-Pieńkowska K, Jamrógiewicz M, Szymkowska K, Krenczkowska D (2016). Direct human contact with siloxanes (silicones)-safety or risk part 1. Characteristics of siloxanes (silicones). Front Pharmacol.

[5] Alhadlaq A, Tang M, Mao JJ (2005). Engineered adipose tissue from human mesenchymal stem cells maintains predefined shape and dimension: implications in soft tissue augmentation and reconstruction. Tissue Eng.

[6] Oliver JD, Eells AC, Saba ES (2019). Alloplastic facial implants: a systematic review and meta-analysis on outcomes and uses in aesthetic and reconstructive plastic surgery. Aesthetic Plast Surg.

[7] Rayess HM, Svider P, Hanba C, Patel VS, Carron M, Zuliani G (2018). Adverse events in facial implant surgery and associated malpractice litigation. JAMA Facial Plast Surg.

[8] Metzinger SE, McCollough EG, Campbell JP, Rousso DE (1999). Malar augmentation: a 5-year retrospective review of the silastic midfacial malar implant. Arch Otolaryngol Head Neck Surg.

[9] Sciaraffia CE, Ahumada MF, Parada FJ, Gonzalez E, Prado A (2018). Bone resorption after use of silicone chin implants, long-term follow-up study with lateral chin radiography. Plast Reconstr Surg Glob Open.

[10] Aiache AE (1992). Mandibular angle implants. Aesthetic Plast Surg.

[11] Al-Jandan B, Marei HF (2018). Mandibular angle augmentation using solid silicone implants. Dent Med Probl.

[12] Vuyk HD (1996). Augmentation mentoplasty with solid silicone. Clin Otolaryngol Allied Sci.

[13] Yaremchuk MJ, Doumit G, Thomas MA (2011). Alloplastic augmentation of the facial skeleton: an occasional adjunct or alternative to orthognathic surgery. Plast Reconstr Surg.

[14] Aynehchi BB, Burstein DH, Parhiscar A, Erlich MA (2012). Vertical incision intraoral silicone chin augmentation. Otolaryngol Head Neck Surg.

[15] Pitanguy I, Martello L, Caldeira AM, Alexandrino A (1986). Augmentation mentoplasty: a critical analysis. Aesthetic Plast Surg.

[16] Hopping SB, Joshi AS, Tanna N, Janjanin S (2010). Volumetric facelift: evaluation of rhytidectomy with alloplastic augmentation. Ann Otol Rhinol Laryngol.

[17] Saleh HA, Lohuis PJ, Vuyk HD (2002). Bone resorption after alloplastic augmentation of the mandible. Clin Otolaryngol Allied Sci.

[18] Abrahams JJ, Caceres C (1998). Mandibular erosion from silastic implants: evaluation with a dental CT software program. AJNR Am J Neuroradiol.

[19] Matarasso A, Elias AC, Elias RL (1996). Labial incompetence: a marker for progressive bone resorption in silastic chin augmentation. Plast Reconstr Surg.

[20] Polo M (2017). Bone resorption under chin implants: the orthodontist's role in its diagnosis and management. Am J Orthod Dentofacial Orthop.

[21] Wellisz T, Lawrence M, Jazayeri MA, Golshani S, Zhou ZY (1995). The effects of alloplastic implant onlays on bone in the rabbit mandible. Plast Reconstr Surg.

[22] Pearson DC, Sherris DA (1999). Resorption beneath silastic mandibular implants: effects of placement and pressure. Arch Facial Plast Surg.

[23] Niamtu III J (2016). Cosmetic Facial Surgery. https://www.booktopia.com.au/cosmetic-facial-surgery-e-book-joe-niamtu-iii/ebook/9780323394024.html.

[24] Sykes JM (2009). Complications of Facial Implants. Complications in Head and Neck Surgery.

[25] Rubin JP, Yaremchuk MJ (1997). Complications and toxicities of implantable biomaterials used in facial reconstructive and aesthetic surgery: a comprehensive review of the literature. Plast Reconstr Surg.

[26] Peled IJ, Wexler MR (1987). Screw fixation of silicone implants. Ann Plast Surg.

[27] von Szalay L (1992). Fixation of facial implants. Plast Reconstr Surg.

[28] Peled IJ (1994). Temporary fixation of facial implants. Plast Reconstr Surg.

[29] Floyd EM, Eppley B, Perkins SW (2018). Postoperative care in facial implants. Facial Plast Surg.

[30] Silver E, Wu R, Grady J, Song L (2016). Knot security—how is it affected by suture technique, material, size, and number of throws?. J Oral Maxillofac Surg.

[31] Shaw RJ, Negus TW, Mellor TK (1996). A prospective clinical evaluation of the longevity of resorbable sutures in oral mucosa. Br J Oral Maxillofac Surg.

